# Thoracic Spine Fractures with Blunt Aortic Injury: Incidence, Risk Factors, and Characteristics

**DOI:** 10.3390/jcm10225220

**Published:** 2021-11-09

**Authors:** Hai Deng, Ting-Xuan Tang, Liang-Sheng Tang, Deng Chen, Jia-Liu Luo, Li-Ming Dong, Si-Hai Gao, Zhao-Hui Tang

**Affiliations:** 1 Division of Trauma & Surgical Critical Care, Department of Trauma Surgery, Tongji Hospital, Tongji Medical College, Huazhong University of Science and Technology, Wuhan 430030, China; d201981743@hust.edu.cn (H.D.); m201976120@hust.edu.cn (L.-S.T.); d202081897@hust.edu.cn (D.C.); m202076263@hust.edu.cn (J.-L.L.); lmdong@tjh.tjmu.edu.cn (L.-M.D.); 2 Class 1901, School of Medicine, Wuhan University of Science and Technology, Wuhan 430065, China; tangzh45@hust.edu.cn; 3 Department of Cardiothoracic and Vascular Surgery, Tongji Hospital, Tongji Medical College, Huazhong University of Science and Technology, Wuhan 430030, China

**Keywords:** blunt trauma, spine injury, thoracic burst fractures, blunt aortic injury

## Abstract

Background: The coexistence of thoracic fractures and blunt aortic injury (BAI) is potentially catastrophic and easy to be missed in acute trauma settings. Data regarding patients with thoracic fractures complicated with BAI are limited. Methods: The authors conducted a prospective, observational, single-center study including patients with thoracic burst fractures. A multivariate logistic regression model was developed to determine the risk factors of aortic injury. Results: In total, 124 patients with burst fractures of the thoracic spine were included. The incidence of BAI was 11.3% (14/124) in patients with thoracic burst fractures. Among these patients, 11 patients with BAI were missed diagnoses. The main risk factors of BAI were as follows: Injury severity score (OR 1.184; 95% CI, 1.072–1.308; *p* = 0.001), mechanism of injury, such as crush (OR 10.474; 95% CI, 1.905–57.579; *p* = 0.007), flail chest (OR = 4.917; 95% CI, 1.122–21.545; *p* = 0.035), and neurological deficit (OR = 8.299; 95% CI, 0.999–68.933; *p* = 0.05). Conclusions: BAI (incidence 11.3%) is common in patients with burst fractures of the thoracic spine and is an easily missed diagnosis. We must maintain a high suspicion of injury for BAI when patients with thoracic burst fractures present with these high-risk factors.

## 1. Introduction

Traumatic vertebral fractures are common in trauma, accounting for about 15% of all trauma hospitalizations, and they often have serious consequences on the medical, social, and financial status of patients [1,2]. The anatomic morphology and biomechanical features of thoracic vertebra are different from those of cervical- and lumbar vertebrae [3,4,5]. Compared with cervical and lumbar fractures, thoracic fractures are typically caused by high-energy trauma due to the stability provided by the thoracic cage, which includes the associated ligaments, costal cartilages, sternum, and associated ribs [2,3,4]. It is estimated that thoracic vertebral fractures account for 20% of all spinal fractures [5].

Thoracic spine fractures range from compression fractures to more severe injuries, such as burst fractures and fracture-dislocations [5]. These severe injuries often lead to spinal instability, which is associated with a high risk of spinal cord injury and has been shown to directly impact health-related quality of life [6]. Apart from spinal cord injury, thoracic spine fractures associated thoracic injuries include sternal fractures, rib fractures, pulmonary contusion, hemopneumothorax, and aortic injury [4]. These related injuries have a greater impact on mortality than thoracic spine fractures themselves [7]. Among these associated thoracic injuries, the clinical signs and symptoms of blunt aortic injury (BAI) are nonspecific and are easy to be misdiagnosed in an acute trauma setting [8]. According to the literature, for those who survive and were taken to hospital, the estimated rate of mortality is 32%, of which one third died before operative intervention [9]. If the diagnosis is timely, it is reported that 60–80% of patients with BAI who arrive at the hospital alive will survive after receiving definite treatment [8].

Therefore, in cases of aortic injury with a thoracic burst fracture, the fracture is very distracting and makes the diagnosis of a BAI extremely difficult. The critical step in avoiding a missed diagnosis in patients who may have BAI is to recognize patients who are at risk. Understanding the risk factors associated with injury is important to promote early diagnosis. However, this association is poorly studied in the medical literature. Some authors have reported an association between spinal fractures and BAI, but only isolated case reports, autopsy studies, and literature review case series [10,11,12,13,14,15]. No study has attempted to elucidate the risk factors for aortic injury in this patient population. Thus, we performed a prospective study of consecutive thoracic burst fractures treated in Tongji trauma center. The primary outcome of the study is to investigate how many patients with thoracic burst fracture are accompanied with aortic injury, and the secondary outcomes are to evaluate risk factors and predictors for aortic injury in patients with thoracic burst fractures, and to describe the clinical features. Thus, to provide a reference basis for emergency physicians (EPs), acute care surgeons or trauma surgeons in clinical diagnosis and treatment, who are usually the first-contact providers to care for such patients.

## 2. Materials and Methods

### 2.1. Study Design and Patient Selection

A prospective, observational, single-center study was performed at the Tongji Hospital, Wuhan. From March 2016 to October 2021, all patients with traumatic nonpathological thoracic burst fractures treated in Tongji Trauma Center were considered for computed tomography angiography (CTA). Inclusion criteria for this prospective analysis were as follows: (1) age over 18 years; (2) patients with burst fractures defined according to the AO system and Magerl classification [6,16]. During the study period, a total of 124 consecutive patients met the eligibility requirements. All patients underwent CTA to determine the presence of an aortic injury. Patients with any aortic abnormality in the CTA, as noted by two experienced and independent radiologists, were recorded as having evidence of an aortic injury.

The present study was approved by the ethics committee at Tongji Hospital and Tongji Medical College (TJIRB20200720). Patient consent for data collection was obtained from each patient or the patient’s legally authorized representative.

### 2.2. Data Collected and Diagnostic Modalities

Patient characteristic information, such as gender, age, mechanisms of injury, and pre-hospital comorbidities, including smoking status, hyperlipemia, hypertension, diabetes mellitus, osteoproliferation, and atherosclerosis, were collected. BAI was classified according to the Society for Vascular Surgery (SVS) classification system [17]. The spine injuries were classified according to the Thoracolumbar Injury Classification and Severity Score (TLICS) at the most damaged level [18]. The injury profile included the injury severity score (ISS), and abbreviated injury score (AIS) for body region. The neurological status of the patient was determined according to the American Spinal Injury Association (ASIA) classification [19]. Whenever present, neurological deficits were recorded as either positive (grade A to D), or negative (grade E). Multiple level thoracic fractures were defined as vertebral fractures extending beyond a thoracic spine element. The diagnostic criteria for flail chest were established according to the guidelines for the diagnosis and treatment of thoracic traumatism: fracture of three or more consecutive ribs in at least two places or visible paradoxical respiration [20]. The diagnosis of osteoproliferation was based on radiographic images and radiological reports. Other information included associated injuries defined as AIS ≥ 2 and included traumatic brain injury (TBI), cervical spine injury, rib fracture, pulmonary contusion, pneumothorax, hemothorax, hemopneumothorax, lumbar spine injury, abdominal injury, pelvic fracture, and extremity injury.

### 2.3. Study End Points

The primary outcome of this study was to determine the incidence of BAI after thoracic burst fractures. Secondary outcomes were to determine risk factors of BAI in this population and to describe the clinical features.

### 2.4. Statistical Analysis

Data were analyzed with SPSS 22.0 (SPSS Inc., Chicago, IL, USA). Prior to analysis, all data were examined for normality and homogeneity of variance. Descriptive statistics were performed for all variables. Categorical variables were expressed as percentages, and continuous variables were expressed as mean and standard deviation or median and range, when appropriate. A Student t-test and the Mann–Whitney U test were used to compare continuous variables and χ^2^ was used to compare categorical variables.

A multivariable logistic regression analysis was performed to identify risk factors for BAI. All the 124 consecutive patients who met the inclusion criteria were classified into the BAI group and non-BAI group. First, variables that were obviously or potentially related to BAI were selected according to the current literature [21]. The factors differentiating the two groups were first identified by univariate analysis. Age, sex, and covariates with statistical significance (*p* ≤ 0.10) were included in the multivariable logistic regression model. The overall fit of the final model is evaluated using the Hosmer–Lemeshow goodness-of-fit test and the area under the receiver operating characteristic (ROC) curve. The final model expressed the odds ratio and 95% confidence intervals. For all tests, *p* < 0.05 was considered statistically significant.

## 3. Results

### 3.1. Incidence of Aortic Injury in Patients with Thoracic Burst Fractures

From March 2016 to October 2021, there were 124 patients with thoracic burst fractures who fulfilled the criteria for the study. All 124 patients underwent CTA evaluation. Of these patients, 14 patients were found to have a BAI and classified into the BAI group (Table 1). The incidence of BAI was 11.3% (14/124) of all admissions for thoracic burst fractures.

### 3.2. Characteristics of Aortic Injury in Patients with Thoracic Burst Fractures

**Figure 1:**
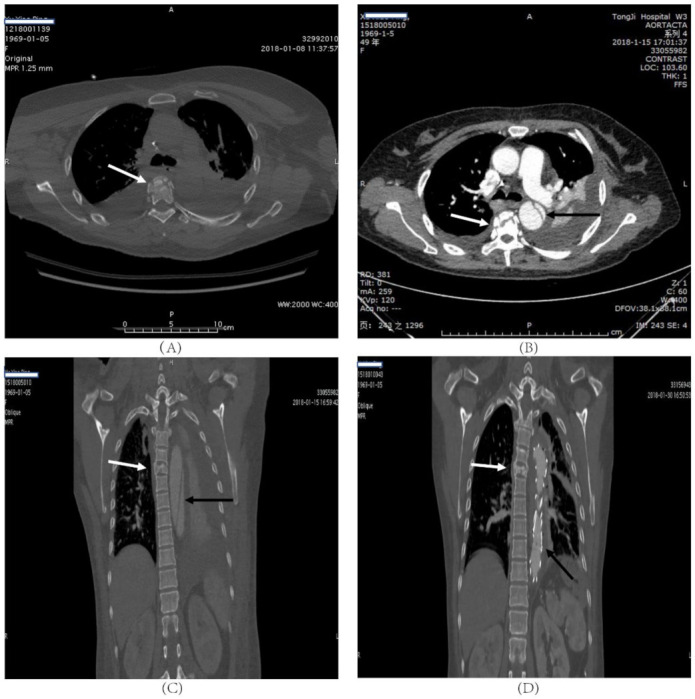
A forty-nine-year-old female who was involved in a chest compression injury. T5 fracture without paraplegia (ASIA D); (**A**) transverse CT scan showed thoracic vertebra fracture (white arrow), and the aortic injury was not obvious. (**B**) Transverse of CT angiography scan of the same patient showed that thoracic vertebra fracture (white arrow) and the aortic injury (black arrow). The aortic injury is obvious on the CT angiography scan. (**C**) Coronal reconstruction of the CT angiography of the same patient showing T5 fracture (white arrow) and formation of thoracic aortic dissection (black arrow). (**D**) Repeat CT of the same patient after the intervention, coronal reconstruction.

**Figure 2:**
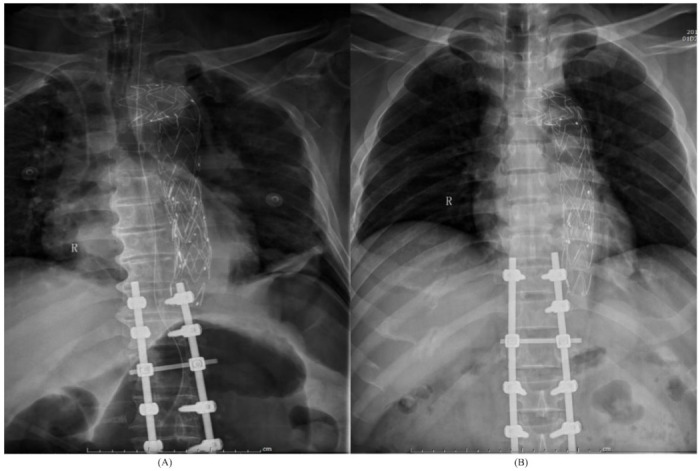
(**A**) A 48-year-old male was injured in a fall, and suffered from multiple injuries, including multiple rib fractures, hemopneumothorax, T11-T12 burst fractures with paraplegia (ASIA A), grade III aortic injury, and with a history of hypertension and hyperlipidemia for 4 years. Post-operative X-rays showing aortic stent-graft in place with posterior instrumentation of the spine. (**B**) A 62-year-old male who was involved in a crush injury and suffered multiple injuries, including multiple rib fractures, pneumothorax, T11-T12 burst fractures with paraplegia (ASIA A), Grade III aortic injury, and with a history of hypertension, hyperlipidemia and atherosclerosis. Post-operative X-rays showing aortic stent-graft in place with posterior instrumentation of the spine.

In the BAI group, there were eleven men and one woman. Representative cases are illustrated in Figure 1 and Figure 2. The mean age of the patients was 48 ± 15 years, the median Injury Severity Score (ISS) was 38 (IQR 29–44.25). The most common mechanism of injury was crush injury (64.3%) (Table 1).

The level of the spine injuries were described as: T11/T12 in four patients, T12 in three, T2/T6/T7/T8 in one, T3/T4 in one, T4 in one, T5 in one, T5/T10 in one, T6-T9/T12 in one, and T7 in one. Of these, six patients had a single affected thoracic level (42.9%) and eight patients (57.1%) sustained multiple levels of injury. Aortic injuries were described as: intimal tear (grade I) in 1 case (7.1%), intramural hematoma (grade II) in 2 (14.3%), and pseudoaneurysm (grade III) in 11 (78.6%). After consultation with vascular surgeons, twelve of these patients underwent endovascular stent-graft to secure the aortic injury. Notably, only three patients admitted to our trauma center immediately after injury were diagnosed with BAI within 3 h after injury. A total of 11 patients with BAI were transferred from a lower-level trauma center due to other serious injuries a few days after injury, and none of them were diagnosed with aortic injury before transferring to Tongji trauma center, in spite of the patients having undergone routine chest CT examination many times after injury in the local hospital. Table 1 summarizes the complete patient demographics.

### 3.3. Risk Factors Analysis

Table 2 shows the comparison between patients with and without BAI. When compared with patients without BAI, those with BAI had a higher ISS (38 [IQR 29–44.25] vs. 27 [IQR18–33]; *p* < 0.01). When compared with those without BAI, patients with BAI were more likely to be injured in a crush injury (64.3% vs. 30.9%; *p* < 0.05), and were more likely to have multilevel thoracic fractures (57.1% vs. 29.1%; *p* < 0.05), neurological deficit (71.4% vs. 38.2%; *p* < 0.05), pulmonary contusion (85.7% vs. 44.5%; *p* < 0.01), lumbar spine injury (57.1% vs. 35.5%; *p* < 0.05), and pelvic fracture (50.0% vs. 17.3%; *p* < 0.01). Additionally, patients with BAI had a higher prevalence of extremity injury compared to those without BAI (50.0% vs. 22.7%; *p* < 0.05). There were no significant differences between BAI and non-BAI group patients in age, sex, TLICS, and mechanism of injury, such as a fall from height.

Univariable analysis of factors associated with BAI is shown in Table 3. The age, sex, mechanism of injury, such as a fall from height, hemothorax, flail chest, history of hypertension and diabetes, and osteoproliferation, were not predictive of BAI. Conversely, ISS, mechanism of injury, such as crush injury, pulmonary contusion, multilevel thoracic fractures, neurological deficit, lumbar spine injury, and extremity injury, were significantly correlated with BAI on univariate analysis (Table 3).

Table 4 shows the results of a multivariable logistic regression of these variables. Injury severity score (odds ratio [OR] 1.184; 95% CI, 1.072–1.308; *p* = 0.001), mechanism of injury, such as crush (odds ratio [OR] 10.474; 95% CI, 1.905–57.579; *p* = 0.007), flail chest (odds ratio [OR] 4.917; 95% CI, 1.122–21.545; *p* = 0.035), and neurological deficit (OR = 8.299; 95% CI, 0.999–68.933; *p* = 0.05) were significantly associated with BAI through multivariate logistic regression analysis.

The accuracy of the model was excellent, and the area under the ROC curve is 0.887. The Hosmer–Lemeshow test showed a good model fit (χ^2^ = 6.729, df = 8, *p* = 0.566).

## 4. Discussion

### 4.1. Summary

Thoracic spine fractures are common in trauma [1,2]. Blunt aortic injury (BAI) is considered as a rare comorbidity in patients with thoracic spine fractures, only reported as a sporadic case [10,11,12,13,14,15]. The coexistence of traumatic thoracic fractures with BAI were potential catastrophic and BAI is an easily missed diagnosis in this case due to lack of understanding of this combined injury. Our study is the first to examine a consecutive series of thoracic burst fractures in a prospective fashion to elucidate the incidence of BAI in patients with thoracic burst fractures. Our study shows that BAI is not uncommon in patients with burst fractures of thoracic spine. In this study, higher risk factors for BAI in thoracic fractures were identified to help guide resource utilization and screen patients. We found that ISS, mechanism of injury, such as crush, concomitant injuries, such as flail chest and neurological deficit, were significantly associated with BAI in patients with thoracic burst fractures.

### 4.2. Incidence of BAI

Overall incidence of BAI among patients suffering from blunt thoracic trauma has been reported to between 1.6% and 2% [4,22]. However, the incidence of BAI was much higher in selected subgroups. In an autopsy study of 304 patients injured due to traffic injuries, aortic injury was found in 102 victims (34%) [14]. James et al., in a retrospective study of 4676 blunt chest trauma patients, found four aortic injuries in 73 patients with thoracic vertebrae (T1 to T8) (5.5%) [15]. In our prospective study of 124 patients with thoracic burst fractures, twelve (11.3%) had BAI confirmed by CTA. In other words, one in every nine patients with thoracic burst fractures has an aortic injury. Our study strongly suggests that patients with thoracic burst fractures are at high risk of aortic injury. Inconsistent with a previous study, we selected only patients with vertebral burst fractures, although patients with other types of spinal fractures may have been at risk for BAI [10]. Since patients with other thoracic injuries were not included, some cases may have not been used and the incidence rate of 11.3% may slightly underestimate the true incidence. The incidence of aortic injury varies differently in different reports, which is most likely attributable to the selection of the patients, small patient cohorts, sensitive and specificity difference in diagnostic methods, and inconsistencies in patient evaluation.

### 4.3. Study Design

While several papers have described the association of spinal fractures with aortic injury, most of them were case reports, autopsy study and literature review case series [10,11,12,13,14,15]. Two retrospective studies reported the correlation between spinal fractures and BAI by first selecting patients with BAI, and then studying these patients for concomitant spine fractures [23,24]. While this does allow for assessing relative risk for BAI according to a specific fracture type, it does not allow for the specification of the risk of associated BAI for this fracture pattern compared with other injuries. Another study has attempted to address the correlation between thoracic fractures and BAI directly by selecting patients with thoracic fractures first, and then evaluating the associated BAI of these patients [15]. However, it was a retrospective study with small numbers, which precluded calculation of relative risks by fracture type.

Although aortography remains the gold standard for the assessment of the thoracic aorta after a blunt trauma, with the increasing popularity and improved accuracy of multidetector computed tomography, CTA has widely replaced aortography as the preferred diagnostic test for BAI in the modern era [22,25]. The sensitivities of reporting CTA range from 95% to 100%, with negative predictive values ranging from 99% to 100% [22]. Therefore, CTA is a justified diagnostic tool for aortic injury in patients with spinal fracture.

In the past two decades, most studies regarding blunt aortic injury have focused on the impact of different treatment approaches, the complication of endovascular aortic repair, diagnostic imaging, and anatomic grading [22,26,27,28]. According to the clinical practice guidelines of the Society for Vascular Surgery, aortic injury was classified into four grades according to its severity: type I (intimal tear), type II (intramural hematoma), type III (pseudoaneurysm), or type IV (rupture) [17]. We grouped all types of aortic injury together into one group. Although the treatment of BAI has been well documented, the risk factors related to BAI are less clear. As such, we focused on the diagnosis and identification of these injuries instead of the treatment, and felt it was reasonable to include all aortic injuries in one group.

### 4.4. Risk Factors

Given the relatively high frequency of thoracic burst fractures with aortic injury, this contrasts with the fact that all aortic injuries in patients with a thoracic burst fracture were missed diagnoses in a lower-level trauma center before being transferred to our trauma center. Although the incidence of thoracic burst fractures with BAI is not low, not all burst fractures of the spine are accompanied by BAI, so these questions remain: which patients with thoracic spine fractures require CTA screening to exclude BAI, and for whom is this an unwarranted expense of money and time? Thus, it is important to understand the factors associated with the injury to promote its early recognition.

The present study showed injuries involved in the crush category are significantly associated with BAI in patients with thoracic burst fractures. In addition, in our analysis, we also found that concomitant injuries, such as flail chest, neurological impairment, and ISS were significantly associated with BAI in patients with thoracic burst fractures. This is certainly reflective of the violence of the injury. Although male sex and older age are risk factors for thoracic aortic dissection [21], our results suggest that sex and age are not independent risk factors for aortic injury after thoracic burst fractures. This is probably due to the particular population of the study. In fact, all patients with thoracic burst fractures—young patients involved in high-energy injuries (for example, traffic accidents) and elderly patients (may have pre-existing vascular diseases) who suffer from low-energy injuries (for example, fall from standing, falling down stairs)—were included.

### 4.5. Mechanism

The mechanism responsible for aortic injury is still not entirely clear. In the literature, it has been suggested that an aortic injury generally occurs either through excessive distraction and stretching of the vessels by C-fracture with antero-posterior dislocation, or through direct trauma to the vessel wall by a fragment of fractured vertebral body [10,29]. A study of the mechanisms of thoracic fractures and associated aortic injury requires a detailed knowledge of the anatomy of the spine and aorta. Normally, the aorta begins to appear in front of the T4 vertebral body and is located more posterolaterally, and the distal descending aorta is located anterior to the vertebrae at the level of diaphragm [30]. In addition, isthmus between the descending aorta and the heart and great vessels make it susceptible to shear forces, particularly from hyperextension injuries at this level [22].

Generally, falls from height are a much more common mechanism than crush in thoracic fractures [6]. In our study, we did not find a statistically increased incidence of aortic injury caused by falling from height. We did find a statistically increased incidence of aortic injury in patients with thoracic burst fractures involved in a crush injury. Multivariate logistic regression demonstrated the odds ratio for crush injury was 10.5, meaning there is an 11 times higher chance of sustaining an aortic injury when thoracic burst fractures occur as a result of a crush injury than from any other injury mechanism. In addition, the odds ratio for flail chest was 4.92, and the odds ratio for neurological deficit was 8.3. Thoracic burst fractures with flail chest and neurological deficit indicated higher energy injury patterns. Due to the special anatomical positional relationship between the thoracic vertebrae and the aorta, the aorta can be forced onto and stretched over the spine, and fracture of vertebral body may exert undue tension and traction on the aorta when the chest is compressed [31]. In addition, as the heart is squeezed between the anterior chest wall and vertebrae, it can force blood from the heart into the aorta, causing a dramatic rise in the blood pressure of the aorta [25,31]. Therefore, the authors have hypothesized that excessive distraction and stretching of the aorta compounded by the rapid increase of intraluminal hydrostatic pressure is the major mechanism of BAI in thoracic burst fractures.

### 4.6. Study Limitations

Our research has several limitations. First of all, the small number of patients with BAI limited the statistic power of the multivariate analysis. Second, our findings may not be transferrable to other trauma centers, due to the recruitment of a single trauma center. Third, our study cannot determine which segment of the spine fracture is more prone to aortic injury. The precise location of these vertebrae fractures may be related to the injury of adjacent aorta [10,15]. Thus, a multi-center, large-sample prospective study will provide the best attainable level of evidence on this issue.

## 5. Conclusions

Specific thoracic fracture patterns confer an increased risk of concomitant BAI. Our results highlight a rate of 11.3% (14/124) for BAI in this specific population. BAI is not uncommon in patients with thoracic burst fractures and is an easily missed diagnosis. In this study, we provide evidence that increased risk of BAI in patients with thoracic burst fractures is associated with flail chest, neurological deficits, ISS, and mechanism of injury, such as crush. Doctors, especially emergency physicians, emergency surgeons, or trauma surgeons who are usually the first contacts to care for such patients, and when patients with thoracic burst fractures have these physical examination or imaging findings, must maintain a high suspicion of BAI.

## Figures and Tables

**Table 1:** Characteristics of patients with aortic injury.

Patient No.	Sex	Age	Mechanism of Injury	Level of Fracture	AO Classification	TLICS	BAI Grade	Neurological Deficit	Pulmonary Contusion	Hemothorax	Flail Chest	Pelvic Fracture	Extremity Injury
1	M	58	Crush	T3/T4	A3/A1	2	III					+	+
2	M	48	Fall	T11/T12	A4/A1	6	III	+	+	+	+		
3	M	45	Fall	T11/T12	A1/A4	6	II	+	+	+	+		+
4	M	55	Crush	T2/T6/T7/T8	A1/A1/A3/A2	2	III		+	+	+		
5	M	54	Crush	T5/T10	A3/A4	5	II	+	+	+	+	+	+
6	M	62	Crush	T11/T12	A1/A3	5	III	+	+		+	+	+
7	M	54	Fall	T12	A3	4	I		+	+	+	+	+
8	M	55	Crush	T7	A3	2	III		+	+	+	+	+
9	F	49	Crush	T5	A3	4	III	+	+	+			
10	M	51	Crush	T6-T9/T12	A1/A1/A1/A3	5	III	+				+	
11	M	57	Other	T11/T12	A4/A3	7	III	+	+	+	+		
12	M	74	Crush	T12	A3	5	III	+	+	+	+		
13	M	18	Fall	T12	A4	4	III		+	+	+	+	+
14	M	63	Crush	T4	A3	2	III		+	+			

TLICS: Thoracolumbar injury classification and severity score; BAI: Blunt aortic injury; TBI: Traumatic brain injury.

**Table 2:** Comparison of BAI and non-BAI patients.

Variable	Non-BAI Patients (*n* = 110)	BAI Patients (*n* = 14)	*p* Value
Age, mean (SD)	48 ± 16	48 ± 15	0.943
Male, *n* (%)	81 (73.6%)	13 (92.9%)	0.115
ISS, median (IQR)	27 (18–33)	38 (29–44.25)	<0.01
Mechanism			
Crush, *n* (%)	34 (30.9%)	9 (64.3%)	0.014
Falls, *n* (%)	58 (52.7%)	4 (28.6%)	0.089
Other, *n* (%)	18 (16.4%)	1 (7.1%)	0.369
Multilevel thoracic fractures	32 (29.1%)	8 (57.1%)	0.035
Neurological deficit	42 (38.2%)	10 (71.4%)	0.018
TLICS	3.5 ± 1.9	4.2 ± 1.7	0.18
Associated thoracic injuries, *n* (%)			
Sternum	14 (12.7%)	4 (28.6%)	0.114
Hemothorax	50 (45.5%)	10 (71.4%)	0.067
Rib fractures	68 (61.8%)	11 (78.6%)	0.219
Pulmonary contusion	49 (44.5%)	12 (85.7%)	0.004
Pneumothorax	10 (9.1%)	1 (7.1%)	0.81
Hemopneumothorax	17 (15.5%)	0	-
Flail chest	7 (6.4%)	3 (21.4%)	0.052
Scapular fracture	17 (15.5%)	1 (7.1%)	0.408
Other injuries (AIS ≥ 2), *n* (%)			
TBI	49 (44.5%)	4 (28.6%)	0.255
Cervical spine injury	23 (20.9%)	1 (7.1%)	0.221
Lumbar spine injury	39 (35.5%)	9 (64.3%)	0.037
Abdominal injury	15 (13.6%)	2 (14.3%)	0.947
Pelvic fracture	19 (17.3%)	7 (50%)	0.005
Extremity injury	25 (22.7%)	7 (50%)	0.029

BAI: blunt aortic injury; ISS: severity of injury score; TLICS: thoracolumbar injury classification and severity score; AIS: Abbreviated Injury Scale; TBI: traumatic brain injury.

**Table 3:** Univariate analysis of risk factors for BAI in patients with thoracic vertebrate fractures.

Risk Factor	OR	95% CI	*p* Value
Age	1.001	0.966–1.038	0.942
Sex	0.215	0.027–1.716	0.147
ISS	1.137	1.059–1.220	<0.001
Crush	4.024	1.254–12.907	0.019
Fall	0.359	0.106–1.213	0.099
Hemothorax	3	0.887–10.149	0.077
Pulmonary contusion	7.469	1.596–34.962	0.011
Multilevel thoracic fractures	3.25	1.044–10.118	0.042
Lumbar spine injury	3.411	1.067–10.898	0.038
Neurological deficit	4.048	1.193–13.733	0.025
Pelvic fracture	4.789	1.504–12.254	0.008
Extremity injury	3.4	1.089–10.616	0.035
Flail chest	4.013	0.906–17.78	0.067
Hypertension	2.84	0.852–9.468	0.089
Diabetes	1.615	0.175–14.918	0.672
Osteoproliferation	0.538	0.141–2.048	0.363
Atherosclerosis	2.044	0.577–7.242	0.268
Alcohol	3.039	0.907–10.185	0.072

ISS: Severity of injury score.

**Table 4:** Multivariable logistic regression model for risk factors of BAI *.

Risk Factor	OR	95% CI	*p* Value
ISS	1.184	1.072–1.308	0.001
Crush	10.474	1.905–57.579	0.007
Flail chest	4.917	1.122–21.545	0.035
Neurological deficit	8.299	0.999–68.933	0.05

* Area under the ROC curve: 0.887; the Hosmer–Lemeshow test: χ^2^ = 6.729, df = 8, *p* = 0.566. BAI: blunt aortic injury; ISS: severity of injury score.

## Data Availability

The data presented in this study are available on request from the corresponding author. The data are not publicly available due to ethical, legal and privacy issues.
